# Analysis of Severe Hypoglycemia Among Adults With Type 2 Diabetes and Nonalcoholic Fatty Liver Disease

**DOI:** 10.1001/jamanetworkopen.2022.0262

**Published:** 2022-02-23

**Authors:** Ji-Yeon Lee, Young-eun Kim, Kyungdo Han, Eugene Han, Byung Wan Lee, Eun Seok Kang, Bong-Soo Cha, Seung-Hyun Ko, Yong-ho Lee

**Affiliations:** 1 Division of Endocrinology, Department of Internal Medicine, Yonsei University College of Medicine, Seoul, Republic of Korea; 2 Department of Statistics and Actuarial Science, Soongsil University, Seoul, Republic of Korea; 3 Division of Endocrinology, Department of Internal Medicine, Keimyung University School of Medicine, Daegu, Republic of Korea; 4 Institute of Endocrine Research, Yonsei University College of Medicine, Seoul, Republic of Korea; 5 Division of Endocrinology and Metabolism, Department of Internal Medicine, College of Medicine, The Catholic University, Seoul, Republic of Korea; 6 Department of Systems Biology, Glycosylation Network Research Center, Yonsei University, Seoul, Republic of Korea

## Abstract

**Importance:**

Previous studies have indicated that liver cirrhosis is associated with hypoglycemia, but there have been no studies investigating the association between nonalcoholic fatty liver disease (NAFLD) and hypoglycemia in noncirrhotic populations with type 2 diabetes.

**Objective:**

To explore the association of NAFLD with severe hypoglycemia among patients with type 2 diabetes.

**Design, Setting, and Participants:**

This nationwide population-based retrospective cohort study using the National Health Insurance System of South Korea included individuals aged 20 years or older who had undergone a medical health examination between January 1, 2009, and December 31, 2012, and were diagnosed with type 2 diabetes. Participants were followed up until December 31, 2015. Data analyses were performed between January 1, 2019, and February 2, 2021.

**Exposures:**

The baseline fatty liver index (FLI) was used as a surrogate marker for NAFLD.

**Main Outcomes and Measures:**

The outcome of interest, severe hypoglycemia, was measured using hospital admission and emergency department visit records with a primary diagnosis of hypoglycemia.

**Results:**

Among 1 946 581 individuals with type 2 diabetes, 1 125 187 (57.8%) were male. During a median (IQR) follow-up of 5.2 (4.1-6.1) years, 45 135 (2.3%) experienced 1 or more severe hypoglycemia events. Participants with severe hypoglycemia, vs those without severe hypoglycemia, were older (mean [SD] age, 67.9 [9.9] years vs 57.2 [12.3] years; *P* < .001) and had lower mean (SD) body mass index (24.2 [3.43] vs 25.1 [3.4]; *P* < .001). Patients with NAFLD tended to have less severe hypoglycemia without consideration of obesity status. However, after adjustment of multiple clinical covariates, including body mass index, there was a J-shaped association between FLI and severe hypoglycemia (5th decile: adjusted hazard ratio [aHR], 0.86; 95% CI, 0.83-0.90; 9th decile: aHR, 1.02; 95% CI, 0.96-1.08; 10th decile: aHR, 1.29; 95% CI, 1.22-1.37), and the estimated risk of hypoglycemia was higher in participants with NAFLD (aHR, 1.26; 95% CI, 1.22-1.30). The association was more prominent in female participants (aHR, 1.29; 95% CI, 1.23-1.36) and those with underweight (aHR, 1.71; 95% CI, 1.02-2.88).

**Conclusions and Relevance:**

In this study, NAFLD was associated with a higher risk of severe hypoglycemia in patients with type 2 diabetes independent of obesity status. Presence of NAFLD should be considered when evaluating vulnerability to hypoglycemia in patients with type 2 diabetes.

## Key Points

**Question:**

Is nonalcoholic fatty liver disease (NAFLD) associated with severe hypoglycemia in individuals with type 2 diabetes?

**Findings:**

In cohort study of more than 1.9 million individuals, participants with type 2 diabetes and NAFLD without cirrhosis had an approximately 26% increased risk of severe hypoglycemia after adjustment for multiple clinical covariates.

**Meaning:**

Knowledge of the association of NAFLD with severe hypoglycemia in adults with type 2 diabetes, independent of obesity status, could help to inform management.

## Teaser

This cohort study explores the association of nonalcoholic fatty liver disease (NAFLD) with severe hypoglycemia among patients with type 2 diabetes.

## Introduction

Hypoglycemia is the most commonly reported adverse effect in the management of diabetes.^1^ Severe hypoglycemia, defined as any hypoglycemia event requiring external assistance for recovery,^2^ is often accompanied by emergency department (ED) visit or hospitalization.^3^ In previous meta-analyses, the pooled prevalence of hypoglycemia is approximately 45% for minor events and 6% for severe events in patients with type 2 diabetes.^4^ Severe hypoglycemia is associated with falls and driving accidents,^5^ dementia,^6^ cardiovascular events, and mortality.^7^ A hypoglycemia experience also increases patients’ fear and distress and lowers psychological health quality.^8^ Considerable costs are associated with its management, ranging from $12 to $1850 per episode.^9,10^ Therefore, the ability to identify individuals at high risk of hypoglycemia is sorely needed.

Older age, kidney insufficiency, and insulin therapy are well known risk factors for hypoglycemia in patients with type 2 diabetes, and Karter et al^3^ recently developed a hypoglycemia risk stratification tool using 6 inputs (age, chronic kidney disease [CKD], insulin use, sulfonylurea use, prior hypoglycemia-related utilization, and prior year ED visit).^3^ Regarding the association between obesity and hypoglycemia, the Action to Control Cardiovascular Risk in Diabetes Study reported that body mass index (BMI [calculated as weight in kilograms divided by height in meters squared]) of 30 or greater was associated with a lower risk of severe hypoglycemia compared with BMI less than 25 (hazard ratio [HR], 0.65; 95% CI, 0.50-0.85).^11^ Additionally, a recent study observed an inverse, J-shaped association between BMI and the development of severe hypoglycemia.^12^

Nonalcoholic fatty liver disease (NAFLD) is a major metabolic liver disease worldwide, and its prevalence, estimated as 25%,^13^ is expected to increase rapidly due to increased prevalence of obesity and aging populations. Because of its association with obesity and insulin resistance, the overall prevalence of NAFLD in patients with type 2 diabetes is reported to be 55.5%.^14,15^ Moreover, NAFLD is an emerging risk factor for various complications, including metabolic syndrome, cardiovascular and kidney diseases, cancers, and overall mortality.^16,17,18,19,20^ However, its association with the development of severe hypoglycemia in patients with type 2 diabetes remains unclear. Therefore, we investigated the association between NAFLD and severe hypoglycemia in patients with type 2 diabetes using a nationwide population-based cohort study.

## Methods

### Data Source

The National Health Insurance Service (NHIS) of South Korea consists of 2 main programs: National Health Insurance for employees and self-employed individuals (97% of the population) and Medical Aid for individuals with low income (the remaining 3% of the population). As a mandatory single insurance organization, the NHIS covers the entire South Korean population of more than 50 million individuals.^21,22^ NHIS records contain demographic characteristics (eg, age, sex, income, residential area), a claims database (consultations, diagnoses by the *International Statistical Classification of Diseases and Related Health Problems, Tenth Revision *[*ICD-10*], and prescriptions), and health check-up data, including anthropometric, laboratory, and questionnaire data (medical history and health behaviors, such as smoking, alcohol drinking, and physical activity).^22,23^ NHIS enrollees are recommended to undergo a general health examination biennially; the participation rate was 74.8% in 2014.^23^

This study was approved by the institutional review board of the Severance Hospital, Yonsei University College of Medicine, and informed consent from study participants was waived due to the retrospective nature of the study. This report followed the Strengthening the Reporting of Observational Studies in Epidemiology (STROBE) reporting guideline for cohort studies.

### Study Population

We evaluated the records of participants aged 20 years or older who had undergone the medical health examination between January 1, 2009, and December 31, 2012. Among them, participants with prevalent type 2 diabetes at baseline and those who developed type 2 diabetes during follow-up were selected. Type 2 diabetes diagnosis was based on either prescription of antidiabetic agents with *ICD-10* diagnosis (E11-E14) in the claims database or fasting plasma glucose level of at least 126 mg/dL on health examination data (to convert glucose to millimoles per liter, multiply by 0.0555). Exclusion criteria were as follows: heavy alcohol consumption (>210 g/week of alcohol consumption for men and >140 g/week for women); hepatitis B or C carrier; a diagnosis of liver cirrhosis (*ICD-10* code K74),^24^ acute or chronic pancreatitis (*ICD-10* codes K85, K86.0, and K86.1), and other diseases of the pancreas (*ICD-10* codes K86.2, K86.3, K86.8, and K86.9); prior diagnosis of pancreas, liver, or biliary tract cancers (*ICD-10* codes C25 and C22) to exclude hypoglycemia by liver and pancreatic disease other than NAFLD; and participants with missing data. A total of 1 946 581 participants with type 2 diabetes were enrolled and followed up until December 31, 2015, for a median (IQR) of 5.2 (4.1-6.1) years (eFigure 1 in the Supplement).

### Clinical and Laboratory Measurements

Demographic and anthropometric data including age, sex, BMI, and waist circumference (WC) were abstracted. Smoking habits were classified as noncurrent or current; alcohol drinking was identified as less than 30 g/day or 30 g/day or greater (alcohol drinker); exercise habit was categorized as less than 3 times/week or 3 times/week or more of vigorous exercise for at least 20 minutes (physically active). Low socioeconomic status was defined as participants with the lowest 20% income status. Blood samples, collected after overnight fasting, included serum glucose, total cholesterol, triglyceride, high-density lipoprotein (HDL) cholesterol, low-density lipoprotein (LDL) cholesterol, liver panel, and γ-glutamyl transferase (GGT) levels as well as kidney function (creatinine level and estimated glomerular filtration rate [eGFR]).

Hypertension was defined as *ICD-10* codes I10-I13 and I15, with antihypertensive drug treatment or systolic/diastolic blood pressure (BP) of 140/90 mm Hg or greater. Hyperlipidemia was identified as *ICD-10* code E78, with lipid-lowering agent prescription or serum total cholesterol level of at least 240 mg/dL (to convert total cholesterol to millimoles per liter, multiply by 0.0259). CKD was considered an eGFR of less than 60 mL/min/1.73 m^2^ using the 4-variable modification of diet in kidney disease formula.^25^ Cardiovascular disease (CVD) was considered as previous heart disease and/or ischemic stroke, which was defined by a combination of *ICD-10* codes (I21-I22, I50 and I63-I64, G458-G459, respectively) and medical histories. Use of antidiabetic drugs (insulin, metformin, sulfonylurea, glinide, thiazolidinedione, and dipeptidyl-dipeptidase 4 [DPP4] inhibitor) was obtained through claims data.

### Definition of NAFLD and Severe Hypoglycemia

The fatty liver index (FLI) was used as a surrogate marker for NAFLD. FLI was calculated using the following formula^26^:

FLI = e^x^ / (1 + e^x^) × 100, where

x = 0.953 × ln(TG) + 0.139 × BMI + 0.718 × ln(GGT) + 0.053 × WC – 15.745,

with triglycerides (TG) measured in millimoles per liter, GGT in U/L, and WC in cm. The index value ranged from 0 to 100. According to a previous study, an FLI score of less than 30 can be used to estimate the absence of fatty liver (sensitivity 87%) and FLI of at least 60 to estimate the presence of fatty liver (specificity 86%).^26^ In this study, participants were classified into 3 groups according to FLI: low FLI (<30); intermediate FLI (30-59); and high FLI (≥60).

The outcome of interest was measured using hospital admission and ED visit records with a primary diagnosis of hypoglycemia (*ICD-10* codes E16.0, E16.1, E16.2, E11.63, E13.63, and E14.63). Previous epidemiologic studies have used hospital admission records to identify severe hypoglycemia events using NHIS data.^12,27^

### Statistical Analysis

Baseline characteristics are presented as either mean (SD) or number (proportion). The HR and 95% CI of severe hypoglycemia events according to FLI was estimated using Cox proportional hazard regression model. Model 1 was unadjusted; model 2 was adjusted for potential confounding factors, such as age, sex, smoking and alcohol habits, exercise, and BMI; model 3 was further adjusted for severe hypoglycemia within the previous 3 years; insulin, sulfonylurea, and/or glinide use; and history of hypertension, CKD, and CVD, which were shown to be associated with the development of severe hypoglycemia (eTable 1 in the Supplement). For example, insulin use was associated with 3.07-fold increased risk of severe hypoglycemia (95% CI, 3.00-3.14). We further analyzed using the Fine-Gray subdistribution hazard regression to account for death as competing risk for severe hypoglycemia.

The number (multiplied by 100 times) of severe hypoglycemia per person during the follow-up period according to FLI was estimated using a generalized linear model adjusted for confounders (as in model 3) and follow-up duration and was reported as the least-square mean (SE). We also examined the association between FLI and severe hypoglycemia events according to age subgroups (<60 or ≥60 years), sex (male or female), BMI (<18.5, 18.5-22.9, 23-24.9, and ≥25), CKD status (yes or no), CVD status (yes or no), insulin use (yes or no), and sulfonylurea and/or glinide use (yes or no). All statistical analyses were conducted using SAS version 9.2 (SAS Institute) and R version 4.1.0 (R Project for Statistical Computing). Statistical significance was set at *P* < .05, and all tests were 2-tailed.

## Results

### Baseline Characteristics of Study Population

Among 1 946 581 individuals with type 2 diabetes, 1 125 187 (57.8%) were men. A total of 45 135 participants (2.3%) experienced at least 1 episode of severe hypoglycemia during a median (IQR) follow-up period of 5.2 (4.1-6.1) years. Participants with severe hypoglycemia were older (mean [SD] age, 57.2 [12.3] years vs 67.9 [9.9] years; *P* < .001) and had lower mean (SD) BMI (25.1 [3.4] vs 24.2 [3.43]; *P* < .001) than participants without severe hypoglycemia. They had a higher rate of comorbidities, such as hypertension, CKD, and CVD, and the proportion of those using insulin, sulfonylurea, and glinides were higher compared with participants without severe hypoglycemia. Baseline characteristics of the study population according to incidence of severe hypoglycemia are summarized in Table 1.

**Table 1.** Baseline Characteristics of Participants

Characteristic	Participants, No. (%)		*P* value
	No hypoglycemia (n = 1 901 446)	Severe hypoglycemia (n = 45 135)	
Demographic parameters			
Age, mean (SD), y	57.2 (12.3)	67.9 (9.9)	<.001
Men	1 104 968 (58.1)	20 219 (44.8)	<.001
Women	796 478 (41.9)	24 916 (55.2)	
Height, mean (SD), cm	162.1 (9.2)	157.5 (9.1)	<.001
Weight, mean (SD), kg	66.2 (11.7)	60.1 (10.4)	<.001
BMI, mean (SD)	25.1 (3.4)	24.2 (3.5)	<.001
Waist circumference, mean (SD), cm	85.2 (8.6)	84.8 (8.9)	<.001
BP, mean (SD), mmHg			
Systolic	128.9 (15.7)	130.9 (17.3)	<.001
Diastolic	79.0 (10.1)	77.7 (10.5)	<.001
Current smoking	465 293 (24.5)	7236 (16.0)	<.001
Current alcohol use	758 761 (39.9)	9543 (21.1)	<.001
Physically active	916 430 (48.2)	15 406 (34.1)	<.001
Low socioeconomic status	515 189 (27.1)	12 841 (28.5)	<.001
Laboratory parameters, mean (SD)			
Fasting glucose, mg/dL	143.5 (43.0)	141.0 (56.7)	<.001
Total cholesterol, mg/dL	197.8 (41.9)	189.8 (43.8)	<.001
Triglycerides, mg/dL	174.5 (117.5)	163.0 (104.1)	<.001
HDL-C, mg/dL	51.3 (16.6)	50.4 (20.0)	<.001
LDL-C, mg/dL	113.0 (37.6)	107.8 (38.3)	<.001
AST, IU/L	27.9 (17.3)	26.1 (17.5)	<.001
ALT, IU/L	30.5 (23.2)	24.5 (18.2)	<.001
GGT, IU/L	48.5 (64.0)	42.2 (72.7)	<.001
Creatinine, mg/dL	1.0 (0.7)	1.1 (0.8)	<.001
eGFR, mL/min/1.73 m^2^	85.1 (35.3)	73.1 (35.8)	<.001
Comorbidities			
Hypertension	1 054 443 (55.5)	34 507 (76.5)	<.001
Dyslipidemia	798 915 (42.0)	21 626 (47.9)	<.001
Chronic kidney disease	204 376 (10.8)	15 359 (34.0)	<.001
Cardiovascular disease	89 642 (6.1)	4395 (10.9)	<.001
Antidiabetic drugs			
Insulin	132 442 (7.0)	12 683 (28.1)	<.001
Metformin	850 459 (44.7)	29 900 (66.3)	<.001
Sulfonylurea	764 336 (40.2)	33 923 (75.2)	<.001
Glinides	38 432 (2.0)	2742 (6.1)	<.001
Thiazolidinedione	116 254 (6.1)	4809 (10.7)	<.001
DPP4 inhibitor	172 405 (9.1)	4177 (9.3)	.17

Abbreviations: ALT, alanine aminotransferase; AST, aspartate aminotransferase; BMI, body mass index (calculated as weight in kilograms divided by height in meters squared); BP, blood pressure; DPP4, dipeptidyl peptidase-4; eGFR, estimated glomerular filtration rate; GGT, γ-glutamyltransferase; HDL-C, high-density lipoprotein cholesterol; LDL-C, low-density lipoprotein cholesterol.

SI conversion factors: To convert ALT, AST, and GGT to microkatals per liter, multiply by 0.0167; creatinine to micromoles per liter, multiply by 88.4; glucose to millimoles per liter, multiply by 0.0555; HDL-C, LDL-C, and total cholesterol to millimoles per liter, multiply by 0.0259; and triglycerides to millimoles per liter, multiply by 0.0113.

### Risk of Severe Hypoglycemia According to FLI

**Figure 1.**
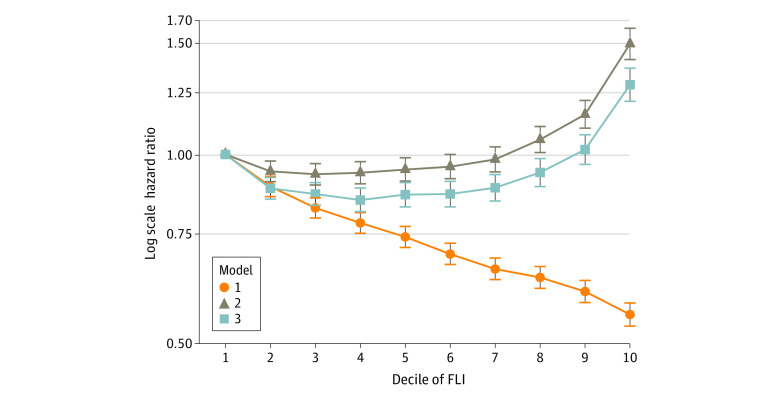
Hazard Ratios for Severe Hypoglycemia According to Fatty Liver Index (FLI) Deciles Model 1 was unadjusted. Model 2 was adjusted for age, sex, smoking and alcohol habits, exercise, and body mass index. Model 3 was further adjusted for severe hypoglycemia within previous 3 years; insulin, sulfonylurea, or glinides use; and history of hypertension, chronic kidney disease, and cardiovascular disease. Error bars indicate 95% CIs.

To assess the association between NAFLD and severe hypoglycemia, HRs for severe hypoglycemia were determined according to FLI deciles (Figure 1). In the unadjusted model (model 1), the risk of severe hypoglycemia gradually decreased with increasing FLI. However, after adjustment for age, sex, smoking and alcohol habits, exercise, and BMI in model 2, the pattern reversed, and there was a J-shaped association between FLI and severe hypoglycemia. This remained after further adjustment for other variables, including severe hypoglycemia within the previous 3 years; insulin, sulfonylurea, and glinide use; and history of hypertension, CKD, and CVD (model 3). The risk of severe hypoglycemia gradually increased from the 5th decile (aHR, 0.86; 95% CI, 0.83-0.90), then sharply increased from the 9th decile (aHR, 1.02; 95% CI, 0.96-1.08) to the 10th decile (aHR, 1.29; 95% CI, 1.22-1.37). The cutoff value of the ninth decile was 83.5 in male individuals and 70.4 in female individuals (eTable 2 in the Supplement).

Although the duration of diabetes may be associated with severe hypoglycemia,^28,29^ the duration of diabetes was not available in this data set, so further analysis was performed only in patients with newly diagnosed type 2 diabetes. Participants with FLI of 60 or greater showed an 88% increased risk of severe hypoglycemia compared with those with FLI of less than 30 (95% CI, 1.67-2.11) (eTable 3 in the Supplement), suggesting the strong association between NAFLD and severe hypoglycemia in newly diagnosed type 2 diabetes.

Next, participants were classified into 3 groups according to FLI: absence of NAFLD (<30), intermediate FLI (30-59), or presence of NAFLD (≥60). The crude incidence rates of severe hypoglycemic events were significantly lower in participants with FLI 30 to 59 and FLI of 60 or greater compared with those with FLI<30 (Table 2). However, in the fully adjusted model, participants with FLI 30 to 59 showed a similar incidence of severe hypoglycemia compared with those with FLI of less than 30 (adjusted HR [aHR], 0.99; 95% CI, 0.97-1.02), whereas participants with FLI of 60 or greater showed a 26% increased risk of severe hypoglycemia compared with those with FLI of less than 30 (aHR, 1.26; 95% CI, 1.22-1.30). The association between higher FLI and severe hypoglycemia was more prominent in women than men (women: aHR, 1.29; 95% CI, 1.23-1.36; men: aHR, 1.17; 95% CI, 1.12-1.23). The Fine-Gray subdistribution HR for severe hypoglycemia for participants with FLI of 60 or greater compared with those with FLI of less than 30 was 1.16 (95% CI, 1.12-1.20) (eTable 4 in the Supplement).

**Table 2.** Association Between Fatty Liver Index and Incident Severe Hypoglycemia Events

Fatty liver index score	Participants, No.		Incident rate per 1000 person-years	HR (95% CI)^a^		
	Incident cases	Person-years		Model 1	Model 2	Model 3
Overall						
<30	22 213	3 880 165	5.7	1 [Reference]	1 [Reference]	1 [Reference]
30-59	14 632	3 310 049	4.4	0.77 (0.76-0.79)	1.03 (1.01-1.06)	0.99 (0.97-1.02)
≥60	8290	2 572 210	3.2	0.56 (0.55-0.58)	1.25 (1.21-1.29)	1.26 (1.22-1.30)
Male participants						
<30	9424	1 775 198	5.3	1 [Reference]	1 [Reference]	1 [Reference]
30-59	6613	1 958 450	3.4	0.64 (0.62-0.66)	0.97 (0.94-1.01)	0.97 (0.94-1.01)
≥60	4182	1 870 224	2.2	0.42 (0.41-0.44)	1.14 (1.08-1.19)	1.17 (1.12-1.23)
Female participants						
<30	12 789	2 104 967	6.1	1 [Reference]	1 [Reference]	1 [Reference]
30-59	8019	1 351 598	5.9	0.98 (0.95-1.00)	1.10 (1.06-1.13)	1.02 (0.99-1.06)
≥60	4108	701 985	5.9	0.96 (0.93-1.00)	1.47 (1.40-1.54)	1.29 (1.23-1.36)

Abbreviation: HR, hazard ratio.

^a^ Model 1 was unadjusted. Model 2 was adjusted for age, sex, smoking and alcohol habits, exercise, and body mass index. Model 3 was further adjusted for severe hypoglycemia within previous 3 years; insulin, sulfonylurea, or glinides use; and history of hypertension, chronic kidney disease, and cardiovascular disease. Values with statistical significance are those for which the 95% CI does not cross 1.

Additionally, the number of severe hypoglycemia episodes according to FLI was examined. Among 1 946 581 participants, 32 652 (1.7%) had 1, 7613 (0.4%) had 2, and 4870 (0.3%) had 3 or more events of severe hypoglycemia. The number (multiplied by 100 times) of severe hypoglycemia per person during follow-up in the group with FLI of 60 or greater was 4.16, significantly higher than 3.57 in the group with FLI of less than 30 and 3.52 in the group with FLI of 30 to 59 group (*P* < .001) (eFigure 2 in the Supplement).

We further examined risk of severe hypoglycemia according to FLI components (eTable 5 in the Supplement). The highest quartiles of GGT and WC were significantly associated with increased risk (GGT: aHR, 1.05; 95% CI, 1.02-1.08; WC: aHR, 1.10; 95% CI, 1.05-1.14). A significantly increased risk of hypoglycemic events was observed in participants with underweight (BMI <18.5) compared with those in the reference range (BMI 18.5-22.9), whereas lower risk was observed in participants with overweight and obesity. There was no significant association between hypertriglyceridemia and risk of severe hypoglycemia.

### Differential Associations of NAFLD With the Risk of Severe Hypoglycemia in Various Subgroups

**Figure 2.**
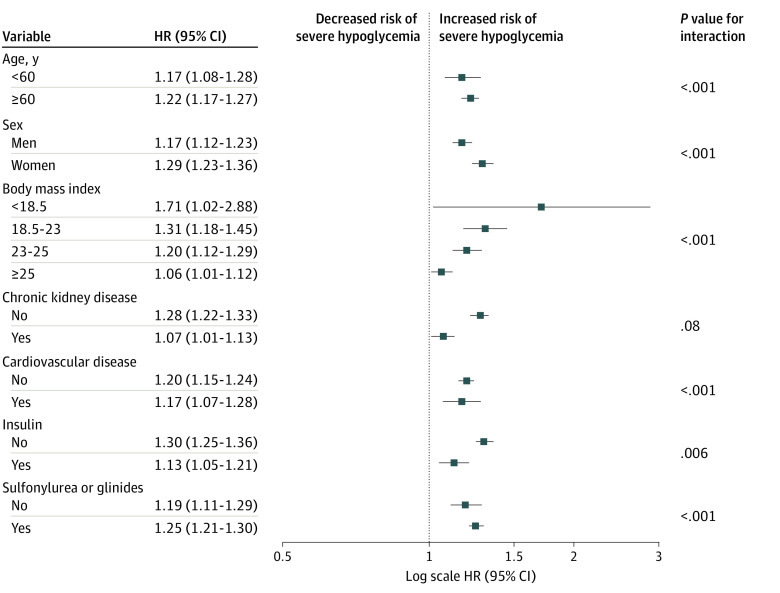
Adjusted Hazard Ratios (HRs) for Severe Hypoglycemia in the Group With Fatty Liver Indices of 60 or Greater vs Those with Fatty Liver Indices of Less Than 0, by Subgroup Cox proportional hazard regression models were used to estimate HRs and 95% CIs. Models were adjusted for age; sex; smoking and alcohol habits; exercise; body mass index (calculated as weight in kilograms divided by height in meters squared); severe hypoglycemia within previous 3 years; insulin, sulfonylurea, or glinides use; and history of hypertension, chronic kidney disease, and cardiovascular disease. Error bars indicate 95% CIs.

The association of NAFLD with severe hypoglycemia was assessed in detailed subgroups by calculating the relative HR in the group with FLI of 60 or greater compared with the group with FLI of less than 30 in a fully adjusted model (Figure 2; eTable 6 in the Supplement). There were significant subgroup differences according to age, sex, BMI, and the use of insulin, sulfonylurea, glinide (eg, participants <60 years: aHR, 1.17; 95% CI, 1.08-1.28; participants ≥60 years: aHR, 1.22; 95% CI, 1.17-1.27; *P *for interaction < .001). The associations between NAFLD and severe hypoglycemia were stronger among participants aged 60 years and older, women, and those who used sulfonylurea or glinide. The associations were most prominent in the lowest BMI (ie, underweight) group (aHR, 1.71; 95% CI, 1.02-2.88). In addition, t he association of degree of NAFLD with the incidence of severe hypoglycemia was lower in those who used insulin compared with those who did not. There was no significant interaction between the subgroup of CKD.

In addition, we performed further analysis using the aspartate aminotransferase/alanine aminotransferase (AST/ALT) ratio (cutoff value, 0.8)^30^ as a surrogate marker for liver fibrosis. Participants were divided into 6 groups according to AST/ALT ratio and FLI, and Cox regression analysis for severe hypoglycemia was performed with the reference group set as those with an AST/ALT ratio of less than 0.8 and an FLI of less than 30. Participants with an AST/ALT ratio of 0.8 or greater had increased risk of severe hypoglycemia compared with the reference group. There was a significant association between NAFLD and severe hypoglycemia risk in groups with fibrosis (eTable 7 in the Supplement). In the fully adjusted model, participants with FLI of 60 or greater and an AST/ALT ratio of 0.8 or greater showed a 38% increased risk of severe hypoglycemia compared with those with an FLI of less than 30 and an AST/ALT ratio of less than 0.8 (aHR, 1.38; 95% CI, 1.31-1.45). This suggests a higher risk of developing severe hypoglycemia in patients with advanced NAFLD.

## Discussion

In this large, population-based longitudinal study, we found that participants with type 2 diabetes and NAFLD had an approximately 26% increased risk of severe hypoglycemia after adjustment for multiple clinical covariates. As lower BMI is known as an independent risk factor for severe hypoglycemia,^11,12,31^ patients with NAFLD seemed to have less risk of hypoglycemia without consideration of BMI. However, after adjusting for BMI, the risk of severe hypoglycemia was significantly increased among participants with NAFLD in a dose-dependent manner. The association of NAFLD with severe hypoglycemia was more prominent in women and in individuals with underweight. These results suggest that clinicians should be aware of the potential for patients with type 2 diabetes and NAFLD to develop severe hypoglycemia.

The liver plays an essential role in glucose production through glycogenolysis and gluconeogenesis, which also serve as defense mechanisms if plasma glucose levels fall below the physiologic range.^32^ In the presence of chronic liver disease, such as liver cirrhosis, glucose metabolism can be dysregulated; previous studies have reported that approximately 30% of patients with liver cirrhosis may have diabetes^33^ and 12% to 16% of patients with both diabetes and cirrhosis had hypoglycemia.^34,35^ However, to our knowledge, there have been no studies investigating the association between NAFLD and hypoglycemia in populations with cirrhosis. We rigorously excluded individuals with liver cirrhosis, those with heavy alcohol use, and hepato-pancreatico-biliary cancers, all of which may affect the occurrence of hypoglycemia. In particular, although we did not diagnose NAFLD by histological or imaging evaluation, the present findings show that patients with an FLI of 60 or greater had significantly higher incidence of severe hypoglycemia than those with an FLI of 30 to 59, indicating a clear dose-dependent association between severity of hepatic steatosis and incidence of severe hypoglycemia in patients with type 2 diabetes.

In our subgroup analyses, participants with NAFLD and a low or even reference range BMI were at greater risk of severe hypoglycemia compared with participants with obesity and NAFLD. Although NAFLD typically occurs in individuals with obesity, a smaller but significant proportion of people develop NAFLD despite a reference range BMI (ie, 25), which is called nonobese NAFLD.^36,37^ The prevalence of nonobese NAFLD is reported as 10% to 20%,^37^ but histologic severity and clinical outcomes compared with patients with obesity and NAFLD are conflicting.^36,37,38^ In this study, we first demonstrated a significant association between nonobese NAFLD and severe hypoglycemia in participants with type 2 diabetes. As lower BMI can reflect malnutrition and coexisting chronic disease,^39^ individuals with a lower BMI might be susceptible to the development of hypoglycemia.

Previous studies have found that older age, prior episodes of hypoglycemia, use of insulin and sulfonylurea, and comorbidities, such as kidney impairment and CVD, are established risk factors for hypoglycemia.^3,40^ Our study results agree with previous results regarding traditional hypoglycemia risk factors (eTable 1 in the Supplement). Also, the present study found that the presence of NAFLD was associated with increased risk of severe hypoglycemia by 1.3-fold, a relatively modest association compared with established risk factors such as insulin or sulfonylurea use and CKD. However, considering the high prevalence of NAFLD in patients with type 2 diabetes, the contribution of NAFLD to hypoglycemia risk is not negligible.

Regarding sex differences in development of hypoglycemia, the previous results are inconsistent. Some studies have reported significantly greater prevalence in women by 1.5- to 1.8-fold,^41,42^ whereas others have not.^31,40^ Our subgroup analyses revealed that female participants with NAFLD were more vulnerable to the development of severe hypoglycemia than their male counterparts. The exact reason is unknown, but hepatic estrogen receptor might have a role in sex differences in hepatic metabolism.^43^ In contrast, those who use insulin were less affected by the presence of NAFLD. As insulin use itself was associated with risk of severe hypoglycemia (aHR, 3.07; 95% CI, 3.00-3.14) (eTable 1 in the Supplement), NAFLD appears to have less additive association.

Possible mechanisms explaining the association of NAFLD with severe hypoglycemia include altered glucose metabolism in NAFLD.^44,45^ Glucagon level is found to be increased in the presence of NAFLD,^46^ and hyperglucagonemia might induce downregulation of hepatic glucagon receptor or blunt the counter-regulatory response to hypoglycemic events in hepatic glucose production.^47^ Also, NAFLD may be associated with glycemic variability through increased oxidative stress,^48,49^ which is an important determinant of hypoglycemia.^50^

### Limitations

This study has limitations. First, we used a previously validated FLI index to define NAFLD^26^ because liver biopsy or imaging was not available in our data set. In the previous study,^51^ FLI showed acceptable accuracy in estimating the presence of steatosis (any histological steatosis ≥5%) in patients with NAFLD. Second, for the outcome of severe hypoglycemia, we were not able to capture events that were asymptomatic or occurred outside the ED or hospital. Additionally, we defined severe hypoglycemia using *ICD* codes but anthropometric or laboratory measurements of diabetes parameters (diabetes duration, serum glucose, glycated hemoglobin) at the time of the event were not available. Third, comorbidities were identified using medical claims data from the NHIS, and coding errors are present in these data sets. Fourth, we were unable to consider the possibility that some patients may progress to more serious disease during the observation period^52^ owing to limited access to the database. Fifth, although, cirrhosis was excluded using the *ICD-10* code, patients with undiagnosed cirrhosis were likely to have been included in the analysis, as NHIS records do not have histological or imaging data of liver. To avoid overestimation and improve diagnostic accuracy, we collected both *ICD-10* codes and prescription or medical questionnaire data. Despite these limitations, this nationwide epidemiologic study found an association between NAFLD and severe hypoglycemia in patients with type 2 diabetes. We analyzed a large historical population cohort of more than 1.9 million patients, which strengthens statistical power and the reliability of results. Also, given that hypoglycemia is common in those with severe alcohol use disorder^53^ and patients with liver disease^54^ because of impairment in gluconeogenesis and glycolysis, we strictly excluded participants with alcohol- or viral-related liver disease and chronic hepato-pancreatic disease.

## Conclusions

In this study, the presence of NAFLD was associated with a 26% increased risk of severe hypoglycemia among participants with type 2 diabetes participants, independent of obesity status. The association was stronger in women and in participants with underweight. This result provides clinicians with additional information about which patients might have a high risk of hypoglycemia to hopefully reduce its incidence and ultimately improve patient safety via individualized therapy. Further validation studies in other racial and ethnic populations and to evaluate causality and mechanisms regarding NAFLD and hypoglycemia risk are warranted.

## Notes

Supplement. **eFigure 1. **Study Flowchart With Data From the Database of National Health Insurance Service **eFigure 2. **Number (Multiplied by 100 ) of Cases of Severe Hypoglycemia per Person During the Follow-up Period According To Fatty Liver Index **eTable 1. **Association Between Traditional Risk Factors and Severe Hypoglycemia Events **eTable 2. **Cutoff Values of Fatty Liver Index Deciles **eTable 3.** Association Between Fatty Liver Index and Incident Severe Hypoglycemia Events in Patients With Newly Diagnosed Type 2 Diabetes **eTable 4.** Fine-Gray Competing Risk Model for Estimating the Subdistribution Hazard Ratio **eTable 5.** Risk of Severe Hypoglycemia by Fatty Liver Index Components **eTable 6.** Adjusted Hazard Ratios for Severe Hypoglycemia in Groups With FLI 60 or Greater and Between 30 and 60 vs Group With FLI Less Than 30, in Detailed Subgroups **eTable 7.** Association Between Fatty Liver Index, AST/ALT Ratio, and Incident Severe Hypoglycemia Events
