# Supplementary material for: Analysis of Severe Hypoglycemia Among Adults With Type 2 Diabetes and Nonalcoholic Fatty Liver Disease
Source: JAMA Netw Open. 2022 Feb 23;5(2):e220262. doi: 10.1001/jamanetworkopen.2022.0262 (PMC8867244; doi:10.1001/jamanetworkopen.2022.0262)
Supplement: Supplement. — eFigure 1. Study Flowchart With Data From the Database of National Health Insurance Service eFigure 2. Number (Multiplied by 100 ) of Cases of Severe Hypoglycemia per Person During the Follow-up Period According To Fatty Liver Index eTable 1. Association Between Traditional Risk Factors and Severe Hypoglycemia Events eTable 2. Cutoff Values of Fatty Liver Index Deciles eTable 3. Association Between Fatty Liver Index and Incident Severe Hypoglycemia Events in Patients With Newly Diagnosed Type 2 Diabetes eTable 4. Fine-Gray Competing Risk Model for Estimating the Subdistribution Hazard Ratio eTable 5. Risk of Severe Hypoglycemia by Fatty Liver Index Components eTable 6. Adjusted Hazard Ratios for Severe Hypoglycemia in Groups With FLI 60 or Greater and Between 30 and 60 vs Group With FLI Less Than 30, in Detailed Subgroups eTable 7. Association Between Fatty Liver Index, AST/ALT Ratio, and Incident Severe Hypoglycemia Events [file jamanetwopen-e220262-s001.pdf]

Supplemental Online Content

Lee JY, Kim YE, Han K, et al. Analysis of severe hypoglycemia among adults with type 2 diabetes and nonalcoholic fatty liver disease. *JAMA Netw Open.* 2022;5(2):e220262. doi:10.1001/jamanetworkopen.2022.0262

eFigure 1. Study Flowchart With Data From the Database of National Health Insurance Service

eFigure 2. Number (Multiplied by 100) of Cases of Severe Hypoglycemia per Person During the Follow-up Period According To Fatty Liver Index

eTable 1. Association Between Traditional Risk Factors and Severe Hypoglycemia Events

eTable 2. Cutoff Values of Fatty Liver Index Deciles

eTable 3. Association Between Fatty Liver Index and Incident Severe Hypoglycemia Events in Patients With Newly Diagnosed Type 2 Diabetes

eTable 4. Fine-Gray Competing Risk Model for Estimating the Subdistribution Hazard Ratio

eTable 5. Risk of Severe Hypoglycemia by Fatty Liver Index Components

eTable 6. Adjusted Hazard Ratios for Severe Hypoglycemia in Groups With FLI 60 or Greater and Between 30 and 60 vs Group With FLI Less Than 30, in Detailed Subgroups

eTable 7. Association Between Fatty Liver Index, AST/ALT Ratio, and Incident Severe Hypoglycemia Events

This supplemental material has been provided by the authors to give readers additional information about their work.

© 2022 Lee JY et al. *JAMA Network Open.*

eFigure 1. Study Flowchart With Data From the Database of National Health Insurance Service

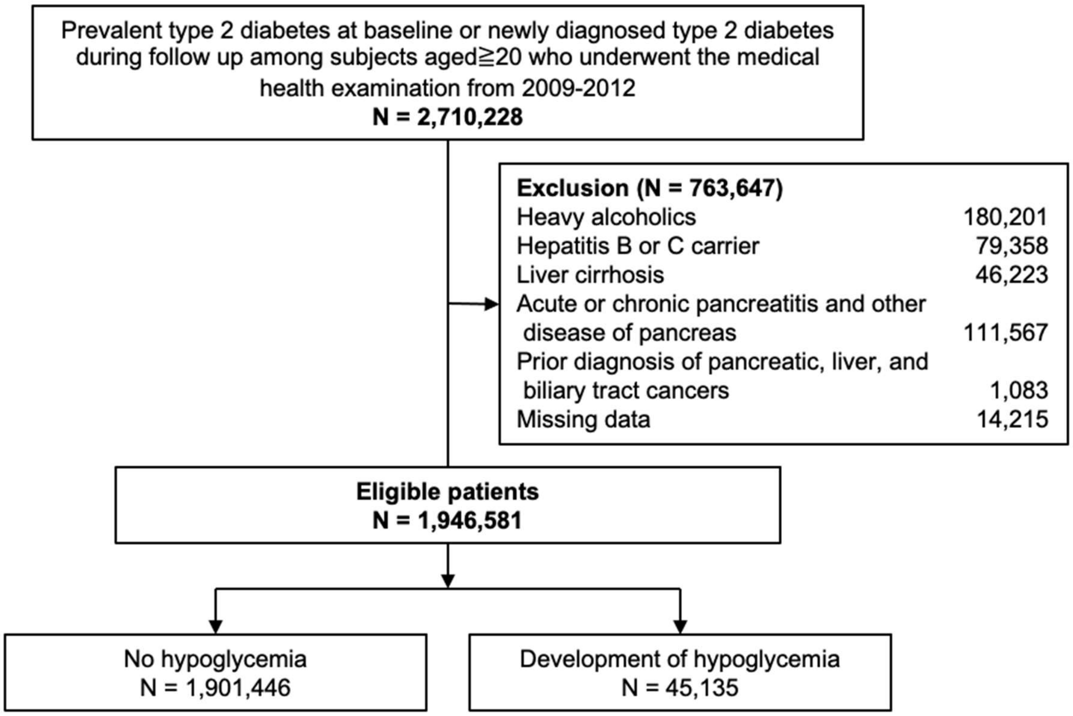

© 2022 Lee JY et al. *JAMA Network Open.*

eFigure 2. Number (Multiplied by 100) of Cases of Severe Hypoglycemia per Person During the Follow-up Period According To Fatty Liver Index

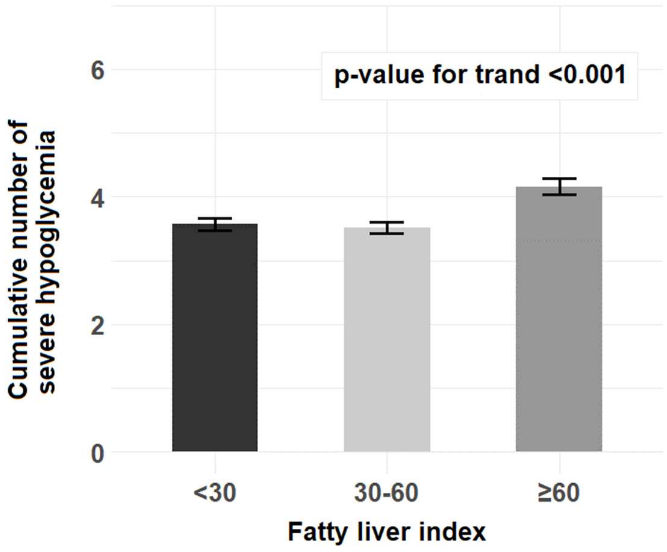

Least-square means and standard errors were adjusted for age, sex, smoking and alcohol habits, exercise, body mass index, severe hypoglycemia in previous 3 years, insulin, sulfonylurea or glinides use, history of hypertension, chronic kidney disease, cardiovascular disease, and follow-up duration. Analyses were conducted in a repeatedly extracted database based on the same inclusion/exclusion criteria during the revision.

© 2022 Lee JY et al. *JAMA Network Open.*

eTable 1. Association Between Traditional Risk Factors and Severe Hypoglycemia Events

Group	Incident cases, n	Person-years, n	Incident rate per 1000 p-y	aHR (95% CI)
**Age, y**				
20-39	501	761,866	0.7	0.75 (0.68-0.82)
40-59	7259	4,522,871	1.6	Ref.
60-69	13,544	2,666,211	5.1	2.01 (1.95-2.07)
70-79	19,612	1,603,974	12.2	3.76 (3.65-6.88)
≥80	4132	206,911	20.0	5.43 (5.20-5.67)
**Sex**				
Male	19,887	5,557,185	3.6	Ref.
Female	25,161	4,204,647	6.0	1.04 (1.01-1.06)
**Insulin use**				
No	32,390	9,072,352	3.6	Ref.
Yes	12,658	689,480	18.4	3.07 (3.00-3.14)
**Sulfonylurea/glinides use**				
No	10,260	5,551,866	1.8	Ref.
Yes	34,788	4,209,966	8.3	1.87 (1.74-2.01)
**Severe hypoglycemia within previous 3 years**				
No	41,535	9,703,353	4.3	Ref.
Yes	3513	58,479	60.1	4.02 (3.87-4.17)
**Hypertension**				
No	10,588	4,327,682	2.4	Ref.
Yes	34,460	5,434,150	6.3	1.30 (1.27-1.33)
**Chronic kidney disease**				
No	29,494	8,630,239	3.4	Ref.
Yes	15,450	1,123,179	13.8	1.89 (1.85-1.93)
**Cardiovascular disease**				
No	34,295	7,123,257	4.8	Ref.
Yes	6294	636,874	9.9	1.12 (1.09-1.15)

Adjusted hazard ratios (aHRs) and 95% CIs were adjusted for age, sex, smoking and alcohol habits, exercise, body mass index, severe hypoglycemia within previous 3 years, insulin, sulfonylurea or glinides use, history of hypertension, chronic kidney disease, and cardiovascular disease

© 2022 Lee JY et al. *JAMA Network Open.*

eTable 2. Cutoff Values of Fatty Liver Index Deciles

Deciles of FLI	Male	Female
D1	<12.3	<7.1
D2	12.3-20.4	7.1-12.1
D3	20.4-28.5	12.1-17.4
D4	28.5-36.7	17.4-23.1
D5	36.7-45.2	23.1-29.5
D6	45.2-54.0	29.5-36.8
D7	54.0-63.2	36.8-45.6
D8	63.2-73.0	45.6-56.2
D9	73.0-83.5	56.2-70.4
D10	≥83.5	≥70.4

FLI, fatty liver index.

© 2022 Lee JY et al. *JAMA Network Open.*

eTable 3. Association Between Fatty Liver Index and Incident Severe Hypoglycemia Events in Patients With Newly Diagnosed Type 2 Diabetes

Group (Fatty liver index)	Incident cases, n	Person-years, n	Incident rate per 1000 p-y	Model 1	Model 2	Model 3
**Overall**						
<30	1713	1,473,352	1.2	Ref.	Ref.	Ref.
30-59	1335	1,316,490	1.0	**0.87 (0.81-0.94)**	**1.34 (1.24-1.46)**	**1.18 (1.07-1.30)**
≥60	1185	1,240,595	1.0	**0.82 (0.76-0.89)**	**2.27 (2.05-2.51)**	**1.88 (1.67, 2.11)**

Model 1 was unadjusted. Model 2 was adjusted for age, sex, smoking and alcohol habits, exercise, and body mass index. Model 3 further adjusted for severe hypoglycemia within previous 3 years, insulin, sulfonylurea or glinides use, history of hypertension, chronic kidney disease, and cardiovascular disease. Values with statistical significance are printed in bold. Analyses were conducted in a repeatedly extracted database based on the same inclusion/exclusion criteria during the revision.

© 2022 Lee JY et al. *JAMA Network Open.*

eTable 4. Fine-Gray Competing Risk Model for Estimating the Subdistribution Hazard Ratio

Group (Fatty liver index)	Model 1	Model 2	Model 3
**Overall**			
<30	Ref.	Ref.	Ref.
30-59	**0.77 (0.75-0.79)**	1.02 (1.00-1.05)	0.97 (0.95-1.00)
≥60	**0.58 (0.57-0.60)**	**1.22 (1.18-1.27)**	**1.16 (1.12-1.20)**

Model 1 was unadjusted. Model 2 was adjusted for age, sex, smoking and alcohol habits, exercise and BMI. Model 3 further adjusted for severe hypoglycemia within previous 3 years, insulin, sulfonylurea or glinides use, history of hypertension, chronic kidney disease, and cardiovascular disease. Values with statistical significance are printed in bold. Analyses were conducted in a repeatedly extracted database based on the same inclusion/exclusion criteria during the revision.

© 2022 Lee JY et al. *JAMA Network Open.*

eTable 5. Risk of Severe Hypoglycemia by Fatty Liver Index Components

Group	Incident cases, n	Person-years, n	Incident rate per 1000p-y	aHR (95% CI)
**Gamma GT Quartile**				
1	15,472	2,533,735	6.1	Ref.
2	10,950	2,366,361	4.6	0.91 (0.89-0.93)
3	9736	2,448,823	4.0	0.91 (0.88-0.93)
4	8977	2,413,505	3.7	1.05 (1.02-1.08)
**Waist circumference Quartile**				
1	11,347	2,368,339	4.8	Ref.
2	10,872	2,515,384	4.3	0.89 (0.86-0.92)
3	10,671	2,381,082	4.5	0.92 (0.89-0.95)
4	12,245	2,497,619	4.9	1.10 (1.05-1.14)
**Body mass index, kg/m^2**				
<18.5	1646	139,950	11.8	1.71 (1.62-1.80)
18.5-22.9	15,291	2,453,462	6.2	Ref.
23-24.9	11,120	2,486,748	4.5	0.75 (0.73-0.77)
25-29.9	14,624	3,960,684	3.7	0.65 (0.64-0.67)
≥30	2454	721,580	3.4	0.68 (0.65-0.71)
**Triglyceride, mg/dL**				
<150	25,704	5,177,760	5.0	Ref.
≥150	19,431	4,584,664	4.2	1.00 (0.98-1.02)

Adjusted hazard ratios (aHRs) and 95% CIs were adjusted for age, sex, smoking and alcohol habits, exercise, body mass index, prior history of severe hypoglycemia within the 3 years, insulin or sulfonylurea or glinides use, history of hypertension, chronic kidney disease, and cardiovascular disease.

© 2022 Lee JY et al. *JAMA Network Open.*

eTable 6. Adjusted Hazard Ratios for Severe Hypoglycemia in Groups With FLI 60 or Greater and Between 30 and 60 vs Group With FLI Less Than 30, in Detailed Subgroups

Group	FLI <30 (ref.)	30≤ FLI <60	FLI ≥60	p-value for interaction
**Age, y**				
<60	Ref.	0.90 (0.84-0.96)	1.17 (1.08-1.28)	**<0.001**
≥60	Ref.	1.02 (0.99-1.04)	1.22 (1.17-1.27)	
**Body mass index, kg/m²**				
<18.5	Ref.	1.17 (0.87-1.55)	1.71 (1.02-2.88)	**<0.001**
18.5-23	Ref.	1.07 (1.02-1.12)	1.31 (1.18-1.45)	
23-25	Ref.	1.02 (0.98-1.07)	1.20 (1.12-1.29)	
≥25	Ref.	0.94 (0.90-0.99)	1.06 (1.01-1.12)	
**Chronic kidney disease**				
No	Ref.	1.01 (0.98-1.04)	1.28 (1.22-1.33)	0.076
Yes	Ref.	0.97 (0.92-1.01)	1.07 (1.01-1.13)	
**Cardiovascular disease**				
No	Ref.	1.00 (0.97-1.02)	1.20 (1.15-1.24)	**<0.001**
Yes	Ref.	0.97 (0.91-1.03)	1.17 (1.07-1.28)	
**Insulin**				
No	Ref.	0.99 (0.96-1.01)	1.30 (1.25-1.36)	**0.006**
Yes	Ref.	1.01 (0.95-1.06)	1.13 (1.05-1.21)	
**Sufonylurea/Glinides**				
No	Ref.	1.04 (1.01-1.07)	1.19 (1.11-1.29)	**<0.001**
Yes	Ref.	0.94 (0.90-0.99)	1.25 (1.21-1.30)	

Fully adjusted for age, sex, smoking and alcohol habits, exercise, body mass index, prior history of severe hypoglycemia within the 3 years, insulin or sulfonylurea or glinides use, history of hypertension, chronic kidney disease, and cardiovascular disease. Values with statistical significance are printed in bold.

© 2022 Lee JY et al. *JAMA Network Open.*

eTable 7. Association Between Fatty Liver Index, AST/ALT Ratio, and Incident Severe Hypoglycemia Events

AST/ALT ratio	Group (Fatty liver index)	Incident cases, n	Person-years, n	Incident rate per 1000 p-y	Model 1	Model 2	Model 3
**< 0.8**	< 30	2529	550000	4.6	Ref.	Ref.	Ref.
	30 - 59	2554	947503	2.7	**0.59 (0.56-0.62)**	**0.81 (0.77-0.86)**	**0.86 (0.81-0.91)**
	≥ 60	1955	1082272	1.8	**0.40 (0.37-0.42)**	**0.91 (0.86-0.97)**	0.99 (0.92-1.06)
**≥ 0.8**	< 30	19839	3309185	6.0	**1.31 (1.25-1.36)**	**0.93 (0.89-0.97)**	**1.05 (1.01-1.10)**
	30 - 59	11857	2312437	5.1	**1.12 (1.07-1.17)**	1.01 (0.97-1.05)	**1.07 (1.02-1.12)**
	≥ 60	6194	1366780	4.5	0.99 (0.94-1.04)	**1.32 (1.25-1.39)**	**1.38 (1.31-1.45)**

Model 1 was unadjusted. Model 2 was adjusted for age, sex, smoking and alcohol habits, exercise and BMI Model 3 further adjusted for severe hypoglycemia within previous 3 years, insulin, sulfonylurea or glinides use, history of hypertension, chronic kidney disease, and cardiovascular disease. Values with statistical significance are printed in bold. Analyses were conducted in a repeatedly extracted database based on the same inclusion/exclusion criteria during the revision.

© 2022 Lee JY et al. *JAMA Network Open.*
